# The Influence of Kindergarten Environment on the Development of Preschool Children’s Physical Fitness

**DOI:** 10.3390/ijerph21060761

**Published:** 2024-06-12

**Authors:** Alice Haav, Leila Oja, Jaanika Piksööt

**Affiliations:** National Institute for Health Development, 10617 Tallinn, Estonia; alice.haav@tai.ee (A.H.); leila.oja@tai.ee (L.O.)

**Keywords:** physical development, physical fitness, preschool childcare institutions, teacher of physical education, learning environment

## Abstract

The aim of this research is to find out to what extent the special qualifications of physical education teachers and the physical environment of kindergartens influence the physical development of preschoolers. Forty-four kindergartens across Estonia participated in the study, half of which had a physical education teacher (PEt), whereas the remaining 22 kindergartens were taught by non-qualified kindergarten teachers (NoPEt). Six Eurofit fitness tests were used to assess the physical development of children (*n* = 704; aged 6–7 years old, with an average age of 6.55 ± 0.5 years). An analysis of variance was used to compare the mean values of the fitness test results of the two groups. Linear regression analysis was applied to clarify the influence of individual and environmental factors on children’s fitness scores. In kindergartens where the position of a PEt had been created, the results of children’s physical fitness were statistically significantly better, more specifically in handgrip strength (m = 12.0, 95% CI = 11.8–12.3 vs. m = 11.5, 95% CI = 11.2–11.7) and in speed tests (m = 23.0, 95% CI = 22.8–23.2 vs. m = 23.6, 95% CI = 23.3–23.8). According to the teacher interviews, these kindergartens also had more rooms and areas specially created for physical exercises. The study revealed that the physical development of children is, when controlling for other individual and environmental factors, influenced by the professional qualification of the PE teacher (95% CI = 0.06–0.56) as well as children’s participation in sports training (95% CI = 0.29–0.83). These findings are important for preschool institutions and municipalities in designing the optimal physical environment for facilitating children’s physical fitness.

## 1. Introduction

Inadequate physical fitness and lack of activity among children is a growing public health problem that manifests itself in younger and younger children. Child growth and maturation dominate the daily lives of children and adolescents for approximately the first two decades of their lives, and these processes can be influenced by physical activity (PA) and fitness [1]. For children and young people, besides PA, another important component of ensuring age-appropriate physical development is also physical fitness, which is also understood as a strong and consistent marker of young people’s health [2].

Studies have proven that there is increasing evidence that physical fitness strongly predicts individual health perspectives [3], being one of the main indicators of health status in both adulthood and childhood [4,5]. As physical fitness is a powerful health marker for children and adolescents, there is no reason to believe that fitness is less important for preschoolers [6]. Previous systematic reviews have demonstrated positive relationships between PA, fitness, cognition, and academic achievement [7]. The first systematic studies on the assessment and analysis of the physical fitness and motor development of preschool children were carried out in Estonia almost thirty years ago [8,9,10]. In a longitudinal study, the development of the same children’s physical abilities and the factors affecting them were evaluated starting from kindergarten, in the first grade of school, and when they reached the seventh grade. Studies have shown that certain physical abilities are quite stable; for example, children with a good jumping ability in the first grade were good jumpers in the seventh grade as well [11].

Most Estonian preschoolers attend kindergarten. Therefore, all kindergartens should offer multiple opportunities for the comprehensive development of children, including the promotion of their health and physical development. Compulsory schooling for Estonian children begins at the age of 7. Until 2015, there was a law in Estonia according to which the position of a physical education teacher (PEt) was mandatory in all kindergartens. After that, the position of a PEt became optional. Since the environment of the kindergarten, including the teacher, has a decisive importance in influencing the physical development of children, it is necessary to obtain an overview of the current situation of the environmental conditions of the kindergartens.

The review of the literature revealed that, in addition to physical activity, physical form is also an important component in ensuring the age-appropriate physical development of children and youth [4], which has positive developmental connections between both cognitive and academic success [7]. Since Estonian children’s schooling begins at the age of 7, most preschoolers go to kindergarten, where children are prepared for school. Since an interesting situation has arisen in Estonia, where according to the current system, kindergartens have the right to remove the special physical education teacher position, we are interested in finding out about children falling behind and the environmental conditions of kindergartens due to the existence of a professional physical education teacher.

### 1.1. The Effect of Fitness on the Development of Preschoolers

In terms of movement, population-based health research often focuses on PA and the need to increase it. In the case of children and young people, attention must also be paid to physical fitness and skills in addition to PA. It is known that the motor development of children, especially in preschool and younger school age, is closely related to both physical activity and fitness [12,13]. Cardiorespiratory fitness, musculoskeletal fitness, and motor fitness have been considered the main health-related components in children, of which cardiorespiratory fitness and muscle strength are the leading factors of health [14].

Longitudinal studies have shown that children who were more active at a younger age were also more likely to be more active at an older age, which is very important from the point of view of the early development of an active lifestyle. It has been shown that PA is positively related to several fitness test results, such as in standing long jump, flamingo balance test, 40 m sprint, and 20 m shuttle run test [15]. Children who do not have adequate motor skills are more likely to not be physically active in middle and later childhood and, therefore, do not develop or maintain health-related aspects of physical fitness. A lower level of fitness negatively affects the child’s ability to continue physical activities that require adequate physical fitness and limits the further development of motor skills. Studies have confirmed that physical fitness is related to both PA and motor competence, implying that the relationship between motor skills and fitness is dynamic and changing throughout childhood [16,17]. Therefore, to ensure the independent PA of young people, it is important to develop motor skills at a younger age and to maintain fitness throughout childhood.

As physical fitness is considered an important indicator of a child’s growth, development, and health status [14,18], physical fitness is also related to the child’s cognitive function [9]. A better level of motor skills in early childhood supports children’s cognitive and social development [19,20]. According to earlier studies, those children who achieved better results in test exercises requiring balance and concentration were better in school readiness [9], and academic achievement tests were also positively related to physical fitness and PA [21]. However, previous studies [22,23] have confirmed that higher-level physical skills can only be reached in a developmentally appropriate, inspiring, and challenging growth environment that creates opportunities and supports practicing various skills. Based on various meta-analyses, Robinson and co-authors [12] point out that movement skills need to be taught and reinforced and do not develop automatically over time. The components of physical fitness provide meaningful information about a child’s physical development and growth. Therefore, physical fitness assessment plays an important role in monitoring children’s health and assessing children’s growth and development [24].

It has been irrefutably proven that in the case of children and young people, motor development is closely related to both physical activity and fitness, which also characterizes the child’s health and physical development. A lower level of fitness negatively affects the child’s physical activity and limits the further development of motor skills. However, development does not happen by itself, as proficient physical skills can only be achieved in a developmentally appropriate, inspiring, and challenging environment accompanied by an educational component. Movement skills must be taught and reinforced because they do not develop automatically over time. Therefore, it is in the context of kindergarten that the components of physical fitness provide meaningful information about the child’s physical development and growth.

### 1.2. Assessment of Physical Fitness

Physical fitness refers to the ability of various body systems to work effectively together to perform daily activities and stay healthy and is typically measured through health-related domains and skill-related indicators [14]. These include body composition, cardiovascular fitness, flexibility, muscular endurance, and strength, as well as agility, balance, coordination, strength, reaction time, and speed. The assessment of the physical fitness of children and young people has been considered as an indicator of the health status of adolescents and is therefore important from the point of view of public health [25], while the assessment of physical fitness has become increasingly relevant, precisely because of the global decline in physical fitness and PA among children and adolescents [24]. Children’s fitness testing has been used in several special-level, cross-sectional, longitudinal, and long-term trend studies [26]. 

There are several sets of tests based on which it is possible to assess the level of physical fitness of children and young people. The oldest of them is the Eurofit test set [27], whose reliability and suitability have been confirmed by several high-level analyses [28]. The FitnessGram and the Alpha-fit set of tests have also been widely used among children and young people [29]. The items of the Eurofit test battery were later used and combined into new test sets, which have been validated and used in several further studies, inc. with children already from 5 years of age [3,30]. 

To date, the ALpha fit [31] and Prefit [24,32] test complexes have been adapted to assess the fitness of preschool children. These test complexes contain several similar test exercises that are widely used to assess the aptitude of young children. Specific testing elements such as BMI, standing long jump, handgrip, single leg stance, sit-and-reach, 4 × 10 m shuttle run are widely used. European child health fitness benchmarks [33] and web-based assessment tools have also been developed to assess the fitness of children and young people. 

When assessing the physical fitness of children, the reliability of these tests for the respective age has always been under question. However, earlier studies have validated the suitability of the tests, and the tests used in one of the oldest sets of tests, the Eurofit tests, have proven to be suitable for preschoolers in kindergarten.

### 1.3. Environmental Factors and Children’s Physical Development

The physical environment of a preschool, which includes all indoor and outdoor facilities and exterior surfaces of a preschool, may have great potential to increase children’s PA. However, it is less clear which specific physical environmental factors are associated with children’s PA. From Tonge et al. research (2016) [34], we know that the conditions of the outdoor environment and the size of the play space are positively related to children’s growth. According to Määttä and others (2019) [35], the variety of PA equipment and the terrain of the playground (except for the gravel field) can be beneficial in increasing children’s PA in preschools.

Studies have evaluated the relationship between daily PA and physical fitness testing (strength, agility, and speed), including health-related (muscular strength and aerobic capacity) and skill-related fitness parameters [36]. It is at preschool age that the indoor and outdoor conditions of the kindergarten, including the physical environment in general, have a significant impact on supporting the development of both PA and motor skills. The literature shows that a better level of physical skills can only be achieved in a growth environment suitable for development, which creates opportunities and supports the practice of various skills.

However, the conditions of preschool physical education teaching, including the environment, teacher characteristics, and children’s participation in a sports club, and the contribution of these factors to the development of physical fitness are not known for sure. The results of this study can provide relevant information on physical and environmental conditions, incl. the importance of special qualifications for physical education teachers in the development of preschoolers’ physical fitness.

## 2. Materials and Methods

Every child in Estonia has the right to early childhood education organized by the municipality and based on the national curriculum. 90% of Estonian children attend municipal preschools. In Estonia, compulsory schooling starts at the age of 7, and children in the preparatory group at the kindergarten are 6–7 years old. 

To establish the environmental conditions in kindergartens and assess the physical development of children, a survey was conducted among the kindergarten personnel, and children were tested for physical fitness in May and June 2017. 

A total of 704 children (363 boys and 341 girls) and 85 teachers participated in the study. This cross-sectional research included children aged 6 to 7 with a mean age of 6.55 ± 0.5 from 44 kindergartens in Estonia. There were 317 (45%) participants who were 6 years old, while 385 (55%) children were 7 years old. 

### 2.1. Anthropometric Measures

Standardized procedures were used for the basic anthropometric measurements. Height was measured to the nearest 0.5 cm using a portable stadiometer (SECA 225, Birmingham, UK); weight was measured to the nearest 0.1 kg using a digital portable scale. The average of two measurements was used for both height and weight. Children were measured without wearing shoes. BMI was calculated and recorded as kg/m^2^. The average body mass index of the children participating in the study was 16.5 ± 2.4.

### 2.2. Procedure for Testing Physical Fitness

The components of the fitness tests used in this study were mostly adapted from the Eurofit fitness test battery [27]. Six physical ability tests were used to assess children’s physical development; all these tests have been validated and are based on exercise activity used in physical education. For comparability, such tests have been selected and have also been used in studies of preschool children previously organized in Estonia [9]. 

The test battery includes a standing long jump test, sit-and-reach test, flamingo balance test, handgrip strength test, 10 × 5 m shuttle run test, and sit-up test. Descriptions of the tests used are given below.

1. The standing long jump test assesses lower-limb explosive strength. The child jumped as far as possible off the stand, trying to land with both feet together and maintaining the equilibrium once landed (he/she was not allowed to put his/her hands on the floor). The score was the distance between the last heel mark and the take-off line. Two tries were allowed, and the longest trial was recorded.
2. The sit-and-reach test measures the flexibility of the hamstring. The test is performed with a standard box with a scale on the top. The participant was required to sit with the untested leg bent at the knee; the tested leg was placed straight with the foot placed against the box. In the back-saver sit-and-reach test, only one leg was evaluated at a time. The participant slowly reached forward as far as possible. The back-saver sit-and-reach test is like the traditional sit-and-reach test, except that the measurement is performed on one side at a time, so each side has its individual score. The results are expressed as an average of both sides.
3. The flamingo balance test measures the ability to balance successfully on a single leg. The child must bend his/her free leg backward, grip the back foot with his/her hand on the same side, and stand like this for one minute. The child is given one try to become familiar with the test before. The examination begins, and the number of attempts needed to balance successfully on a single leg over one minute is accounted. Children were excluded if they had to put down their feet 15 times or more within the first 30 s (s). The test score is expressed as the sum of attempts with both feet; lower scores indicate better performance.
4. The purpose of the handgrip strength test is to measure the maximum isometric strength of the hand and forearm muscles. The subject holds the dynamometer. The participant was asked to squeeze a dynamometer (Takei 5401 Digital Dynamometer, Tokyo, Japan) with maximal isometric effort for approximately 5 s. The best score for each hand was selected from two trials and averaged.
5. The 10 × 5 m shuttle run test is a measure of speed and agility. Participants run back-and-forth over 5 m 10 times. The child is instructed to run as quickly as he/she can after the starting signal. Two tries were allowed, and the best score was recorded (in seconds). For the shuttle test, lower scores indicate better performance.
6. The sit-up test is a measure of the endurance of the abdominal and hip-flexor muscles. The aim of this test is to perform as many sit-ups as you can in 30 s.

A total fitness score was calculated based on the test scores above the median result (50th percentile) among boys and girls. The score was calculated in the range of 0–6 points, where the maximum points indicate that the child performed all six tests over the median result. 

### 2.3. Survey Procedure and Sample

According to the Estonian Education Information System, there are 635 preschool institutions in Estonia. At the beginning of the study, 477 of them were in kindergartens where, according to the conditions of the study, there were at least 3 groups of children, including a group of preschoolers. Of these, 334 kindergartens had a physical education teacher, and 143 did not have a physical education teacher. The kindergartens to be invited to the study were randomly selected from five Estonian regions (South, North, West, Central, and Northeast) among all the institutions that met the prerequisites. The sample was representative as the percentage distribution between the five regions in the final sample was the same as in the general population.

The sample of the study included 44 kindergartens across Estonia. In half of them, the position of physical education teacher (PEt) had been created, which was recruited by a qualified PEt. In the remaining 22 kindergartens, physical education was taught by kindergarten teachers without special qualifications (NoPEt). An online survey was conducted to characterize the environmental conditions of kindergartens. The respondents were physical education teachers of preschool groups or those conducting physical education classes if there was no special physical education teacher in the kindergarten. The teacher’s online survey explained the respective qualifications of the teacher giving PE lessons, the number of PE lessons per week, the variety of different physical activities in the learning process, and the availability of learning places (rooms and areas) created for PE teaching and the variety of learning tools in indoor and outdoor conditions.

### 2.4. Data Analysis

One-way ANOVA was used to clarify the differences between children’s fitness test results in kindergartens with a PE teacher and with no PE teacher.

Linear regression analysis was performed using preschool children’s total fitness score—the number of fitness tests (from 0 to 6) that children performed over the 50th percentile among boys or girls—as the dependent variable. Individual (sex, BMI, participation in sports training) and environmental measures (PE teacher in the kindergarten, PE lessons in a week, variety of movement activities in kindergarten, variety of spaces/areas for movement activities, variety of PE equipment in the gym and outdoors) were used as independent variables. Multiple linear regression was performed to clarify the effect of each independent factor on the children’s total fitness score when controlling for all other factors included in the model. As a result, unstandardized regression coefficients (β) and 95% confidence intervals (CI) for β were presented. 

All analyses were considered statistically significant at the level of < 0.05. Data analysis was performed using IBM SPSS Statistics 28 for Windows.

## 3. Results

### 3.1. Personnel

The average age of both the PE teachers and the preschool teachers participating in the survey was more than 40 years, and the average length of service was 17 years. The survey revealed that in PEt kindergartens, 91% of those conducting physical education classes had the PE qualification, while in NoPEt kindergartens, 18.2% were present.

Most of the qualified PE teachers had also completed the corresponding further training in the last five years, whereas only half of the unqualified PE teachers had attended further training. Teachers of PEt kindergartens engage in PA somewhat more often than employees of NoPEt kindergartens.

### 3.2. Physical Fitness of Children

In the kindergartens that had created the PEt position, the results of children’s physical fitness were statistically significantly higher in the hand strength test and in the shuttle run tests, in addition to the total fitness score (Table 1).

The results of linear regression analysis (Table 2) showed that none of the selected kindergarten environmental factors were significantly related to children’s physical fitness, neither the number of hours of physical education per week nor the variety of activities, areas, and equipment. Only the presence of a PE teacher in kindergarten was significantly related. From the individual factors, the total fitness score of preschoolers was positively related to their lower BMI values and participation in sports training.

When controlling for all other individual and environmental variables in the multivariate regression model, only the position of PEt in the kindergarten (95% CI = 0.06–0.56) and children’s participation in sports training (95% CI = 0.29–0.83) were statistically significant. Thus, an interesting result emerges: PE teachers are important in ensuring children’s development, regardless of the differences in the level of equipment or learning activities in kindergartens.

### 3.3. Organization of Physical Education and Environmental Conditions

In kindergartens, there are generally regular classes of PE twice a week, lasting about 30 min. A wide variety of activities are carried out both indoors and outdoors. Most kindergartens offer additional opportunities for children to exercise. More training opportunities have been created in kindergartens with a special position of PE teacher.

PEt kindergartens had more rooms and areas specifically designed for PA. In these kindergartens, both the means and the availability of the necessary rooms and areas were in better condition for conducting PE activities. In most of the kindergartens participating in the survey, changes improving the possibilities for indoor and/or outdoor physical activities of children had occurred in the past ten years (Table 3). In the highest number of cases, the improvements concerned the acquisition of sports equipment, but sports grounds and other exercise areas were built as well.

The kindergartens with a PE teacher position had carried out significantly more changes related to improving the conditions for PA. These kindergartens also had more premises adapted for PA or sports halls. In the opinion of the persons conducting PE classes, the quality level of the areas suitable for physical exercise was slightly over the average (Table 4). The existence of equipment in the opinion of the persons conducting PE classes and the condition of the equipment of the sports hall/exercise premises was generally good. The situation was slightly better in kindergartens with the PE teacher position.

The results of this survey show that the physical development of children is positively influenced by the environment created in the kindergarten, including the existence of suitable indoor and outdoor areas and equipment, the professional background of the teachers of PE, dedicated physical activities conducted by the teachers, as well as children’s additional sports training. 

## 4. Discussion

As the question of our study was to clarify to what extent the physical fitness of preschoolers is influenced by the special qualifications of physical education teachers and the physical environment of kindergartens, then our study revealed that in addition to actively organized extracurricular activities and the choice of sports and exercise equipment, the most important influence on a child’s physical fitness is a skilled physical education teacher, who coordinates all these activities in the kindergarten. Ensuring and monitoring physical fitness in the first years of life should be considered as one of the aspects of primary prevention and health promotion. 

Physical fitness is considered one of the foundations of an active lifestyle in later life, and its levels have direct and indirect effects on health status and disease prevention in adulthood [6]. Previous studies [37,38] have confirmed the strong impact of early childhood education and childcare-based interventions at different levels on the cardiorespiratory fitness of preschool children. The importance of not only increasing PA but also improving physical fitness is increasingly emphasized, according to which both gender-based and developmentally appropriate interventions can increase children’s physical fitness [39], even in the younger preschool age, at the age of 4–5 years, intervention activities show results in promoting children’s fitness [40].

In addition to the interventions, it is expedient to use the already existing teacher resources to ensure children’s development by improving the teachers’ skills and qualifications. According to our study, in kindergartens that had a position for a PE teacher, most of the teachers of PE had the relevant qualifications. However, in kindergartens where there was no special position for a PE teacher, and the PE classes were conducted by other preschool teachers, most of them did not have the relevant PE qualifications. It should be noted that university curricula do not include sufficient preparation for teaching PE in the preparation of kindergarten teachers. This gap is still unfilled. The results of the survey revealed that in kindergartens where the position of physical education instructor has not been created, PE classes are mostly conducted by teachers without special educational training who have not completed any additional training in PE in the last five years.

Institutional factors, such as teacher training, as well as PA equipment and toys installed in classrooms and play areas, are known to be important intervention methods to improve PA in young children [41], and implemented strategies to improve the PA environment allow for a greater effect to children development. The present study confirmed that it is not always enough to have sports equipment both indoors and outdoors; for the physical development of children, skilled guidance from the teacher is also necessary.

As almost all Estonian children aged 6–7 years attend preschool during weekdays, this study supports a growing body of evidence that points to the importance of teacher professionalism in developing children’s physical development. Physical fitness can be considered as an integrated measure of musculoskeletal, cardiorespiratory, psycho-neurological, and endocrine-metabolic status related to daily PA and/or physical exercise. Physical fitness testing can check the child’s functional status, which is why physical fitness is considered one of the most important health markers [14]. The physical fitness of children and adolescents is very important for the healthy development of adulthood in the future. Studies have confirmed that the development of good physical fitness in children and adolescents can effectively reduce the risk of various chronic cardiovascular diseases, high blood pressure, diabetes, and all mortality and can effectively prevent the development of various chronic cardiovascular diseases [42]. Our study showed that in the kindergarten where the PE teacher was a qualified teacher, the children’s physical ability tests were also better in both speed and strength tests.

Based on the results of the survey, it is possible to give a general overview of the differences between the kindergartens that have created a position for a PE teacher and those that have not (see Table 3 and Table 4). As regards practical work, the kindergartens with a PE teacher have introduced more changes aimed at improving the indoor and outdoor conditions to better suit physical exercises, leading to an environment more conducive to physical activities.

Previous studies have observed that higher levels of PA were associated with better physical fitness values [4,43,44], and participation in preschool physical education and participation in sports clubs were also associated with higher arm strength and running speed [36]. Since physical fitness is related to both motor competence and PA, and these connections strengthen with age [17], it is precisely at a young age that greater attention should be paid to the development of fitness. Our study confirmed that children who attended sports training also had better physical fitness results, regardless of the conditions offered in the kindergarten.

Research to date has not informed about which specific environmental factors may be beneficial for increasing children’s PA and improving children’s fitness. It is known that different instruments may have different associations with children’s PA levels. For example, the presence of portable jumping equipment and the presence of a structured track on the playground were associated with higher levels of outdoor PA [45]. Our study confirmed the importance of outdoor play activities in developing children’s fitness. Kindergartens with a PE teacher had more sports fields or practice areas and adapted outdoor areas for children to be active.

## 5. Strength and Limitations

The strength of this work lies in the practical implications of the results. Non-systematic management of physical education does not guarantee the recommended level of physical development for preschool children. The most up-to-date changes in the promotion of children’s physical education require, first, additional training for teachers and more outdoor activities. In preschools where there is a specially qualified physical education teacher, there are better results in children’s physical development, as well as better sports facilities and exercise equipment. These results are important for preschool institutions and municipalities in designing the perfect physical environment for the development of children’s physical fitness.

There are two possible limitations to the interpretation of the research results: administrative feedback on the one hand and family influence on the other. In terms of administrative feedback, we do not have an assessment of the physical education teacher’s work by kindergarten managers. At the same time, we know whether the child participates in a sports club or not, but we do not know how many times a week the child practices, i.e., what the volume of training per week is.

However, the presented limitations do not reduce the important results of the study, according to which it is important to use specially trained PE teachers in educational activities, not only in school but also in kindergarten.

## 6. Conclusions

A qualified physical education teacher not only positively influences children’s fitness development but can also contribute to an institutional environment for physical development and activities. The results of the study show that the physical development of children in the kindergarten environment is positively influenced by the presence of suitable indoor and outdoor areas and equipment for movement, events conducted by the PE teacher, as well as children’s participation in additional sports training, but the most important of these is the professional background of PE teachers. Important aspects regarding the teachers are also their participation in the relevant in-service training, teachers’ awareness of children’s PA, and teachers’ own PA. The outcomes of the study have an important practical value, showing that the PE qualifications of preschool teachers have a significant impact on supporting children’s physical fitness.

The main conclusion of the study was that differences in physical fitness tests were more related to the presence of a physical education teacher in the kindergarten and children’s participation in sports training, regardless of the good PE environmental conditions of the kindergartens or the length of the physical education class.

## Figures and Tables

**Table 1:** The mean results of preschool children (*n* = 704) fitness tests in kindergartens with PE teacher and with no PE teacher.

Fitness Test	Mean Score (95% CI)		F ^a^	Sig. ^a^	Total		The 50th Percentile	
	PEt	NoPEt			m (95% CI)	Min–Max	Boys	Girls
Sit-and-reach (cm)	20.9 (20.4–21.5)	20.7 (20.2–21.3)	0.21	0.64	20.8 (20.4–21.2)	6–37	19.0	22.5
Handgrip strength (kgf)	12.0 (11.8–12.3)	11.5 (11.2–11.7)	**7.61**	**0.006**	11.8 (11.6–12.0)	4.5–20.5	12.5	11.0
Standing broad jump (cm)	123.6 (121.9–125.2)	122.4 (120.4–124.3)	0.87	0.35	123.0 (121.7–124.3)	67–180	126.0	118.0
10 × 5 m shuttle run (s)	23.0 (22.8–23.2)	23.6 (23.3–23.8)	**14.15**	**0.000**	23.3 (23.1–23.4)	17.0–36.8	22.8	23.3
Flamingo balance (n/60 s)	2.1 (1.9–2.3)	2.1 (1.9–2.3)	0.06	0.81	2.1 (2.0–2.3)	1–13	2	1
Sit-ups (n/30 s)	11.6 (11.2–12.0)	11.4 (11.0–11.8)	0.45	0.50	11.5 (11.2–11.8)	1–25	12	11
Total fitness score ^b^	3.4 (3.3–3.6)	3.1 (2.9–3.2)	**8.72**	**0.003**	3.3 (3.1–3.4)	0–6	3	3

^a^ One-Way ANOVA, statistically significant differences are marked in **bold**; ^b^ The number of fitness tests (from 0 to 6) that children performed over the 50th percentile among boys or girls.

**Table 2:** The influence of individual and environmental factors on preschool children’s (*n* = 704) physical fitness ^a^ according to linear regression analysis.

Model Variables	Univariate Model			Multivariate Model ^b^		
	β	95% CI	Sig. ^c^	β	95% CI	Sig. ^c^
Individual factors						
Sex	0.02	−0.22–0.27	0.85	0.08	−0.16–0.33	0.50
BMI	−0.06	−0.11–−0.01	**0.03**	−0.05	−0.10–0.00	0.06
Participation in sports training	0.58	0.33–0.84	**<0.001**	0.56	0.29–0.83	**<0.001**
Kindergarten environment						
PE teacher in the kindergarten	0.36	0.12–0.61	**0.003**	0.31	0.06–0.56	**0.02**
PE lessons in a week (minutes)	−0.00	−0.01–0.01	0.73	0.00	−0.00–0.01	0.58
Variety of movement activities in kindergarten	0.05	−0.04–0.13	0.27	0.04	−0.06–0.13	0.44
Variety of spaces/areas for movement activities	−0.00	−0.02–0.02	0.92	−0.02	−0.05–0.01	0.19
Variety of PE equipment in the gym and outdoors	0.00	−0.00–0.01	0.17	0.00	0.00–0.01	0.06

^a^ The number of fitness tests (from 0 to 6) that children performed over the 50th percentile among boys or girls; ^b^ Adjusted to all variables in the model; ^c^ Statistically significant values are marked in **bold**.

**Table 3:** Changes made in conditions of the kindergartens’ indoor and outdoor environment during recent years in kindergartens with PE teacher and with no PE teacher.

Changes Made in Connection with PE	PEt ^a^	NoPEt ^a^
Acquisition of new sports equipment, diversification	13	10
Construction of sports fields, exercise areas, stadiums, running tracks, etc	12	6
Adapting and supplementing the outdoor area with new and different sports equipment	8	4
Improving and renovating the condition of the hall or gymnasium	6	4
Adjusting the daily schedule for daily outdoor activities	2	–
Construction of a new kindergarten, complete renovation of the house	–	2
Construction of new sports grounds near the kindergarten, their use	2	–
Adapting new forms of movement to kindergarten PE activities (e.g., orienteering, yoga)	1	1
Total	44	27

^a^ The number of kindergartens where the changes have been made (Max = 22).

**Table 4:** Rooms and areas adapted for physical education and physical activity, and assessment on their condition in kindergartens with PE teacher and with no PE teacher.

Rooms and Areas Adapted for PE and PA	PEt		NoPEt ^b^	
	Number ^a^	Condition ^b^	Number ^a^	Condition ^b^
1. A hall where music lessons also take place	21	4.2	20	4.2
2. A room or gym specially adapted for PE classes	15	4.3	8	4.8
3. Group rooms	21	3.4	22	3.6
4. Swimming pool	4	4.8	5	4.8
5. An outdoor area specially designed to support physical activity	22	3.6	18	3.8
6. Outdoor area with a spacious lawn where children can run freely	23	4.1	22	4.2
7. PA activity area installed in group rooms	9	2.4	4	3.3
Total average score (min–max)	23	19.1 ^c^ (9–30)	22	18.1 ^c^ (7–26)

^a^ The number of kindergartens (Max = 22); ^b^ Condition of the rooms and areas (1–5 point scale); ^c^ The total score for the seven areas varies from 1 to 35, with an average score of 17.5. A score of 35 indicates that the condition of all seven rooms or areas is very good. In other words, the higher the score, the more diverse and better the opportunities for PA in the kindergarten.

## Data Availability

The data presented in this study are available on request from the corresponding author.
